# Association Between Visual Acuity and Incident Atherosclerotic Cardiovascular Disease: A Longitudinal Test of Mediators

**DOI:** 10.5334/gh.1406

**Published:** 2025-02-24

**Authors:** Zijing Du, Xiayin Zhang, Gabriella Bulloch, Feng Zhang, Yu Huang, Yaxin Wang, Yingying Liang, Guanrong Wu, Zhuoting Zhu, Xianwen Shang, Yijun Hu, Xiaohong Yang, Honghua Yu

**Affiliations:** 1 Guangdong Eye Institute, Department of Ophthalmology, Guangdong Provincial People’s Hospital (Guangdong Academy of Medical Sciences), Southern Medical University, Guangzhou, China; 2 Centre for Eye Research Australia, Royal Victorian Eye and Ear Hospital, East Melbourne, VIC, Australia; 3 Department of Ophthalmology, Linyi People’s Hospital, Linyi 276003, Shandong, China; 4 Experimental Ophthalmology, The Hong Kong Polytechnic University, Hong Kong, People’s Republic of China; 5 Guangdong Provincial Key Laboratory of Artificial Intelligence in Medical Image Analysis and Application, Guangdong Provincial People’s Hospital, Guangdong Academy of Medical Sciences, Guangzhou 510080, China

**Keywords:** Visual acuity, atherosclerotic cardiovascular disease, mediators, older adults, longitudinal

## Abstract

**Background:**

Little is known about the prospective relationship between visual acuity (VA) and atherosclerotic cardiovascular disease (ASCVD) events and the extent to which this association is mediated via potential mediators. This study aims to investigate the relationship between VA and ASCVD events, including the mediation effects of potential factors.

**Methods:**

A prospective study was conducted using data from 110,522 participants in the UK Biobank, all of whom had baseline visual acuity (VA) measurements collected between 2006 and 2010. VA was assessed using the logarithm of the minimum angle of resolution (logMAR) chart, with the better-seeing eye selected for analysis. Incident ASCVD events were obtained from hospital admissions and death records up to April 2021. The longitudinal association between VA and ASCVD was examined using Cox proportional hazards models. A four-way decomposition mediation analysis was performed to quantify the indirect effects of hypertension, diabetes, depression, and socioeconomic status in mediating the relationship between VA and ASCVD.

**Results:**

Over an 11.13-year median follow-up, 5,496 ASCVD cases were recorded. A one-line worsening in VA (0.1 logMAR increase) was associated with an increased risk of ASCVD (HR = 1.63; 95%CI = 1.35–1.96, *P* < 0.001). Mediation analysis showed that hypertension, diabetes, depression, and Townsend deprivation index contributed 3.8%, 3.3%, 5.7%, and 5.9% to this association, respectively (all *P* < 0.05). Notably, depression was the strongest mediator, accounting for 10.0% of the association in women (*P* < 0.05).

**Conclusions:**

Our study demonstrates that visual decline is associated with an increased risk of ASCVD. Early intervention through regular eye exams can help mitigate the risk of ASCVD in middle-aged and older adults. Additionally, mental health is a key mediator in the VA-ASCVD relationship, particularly among women.

**Graphical Abstract:**
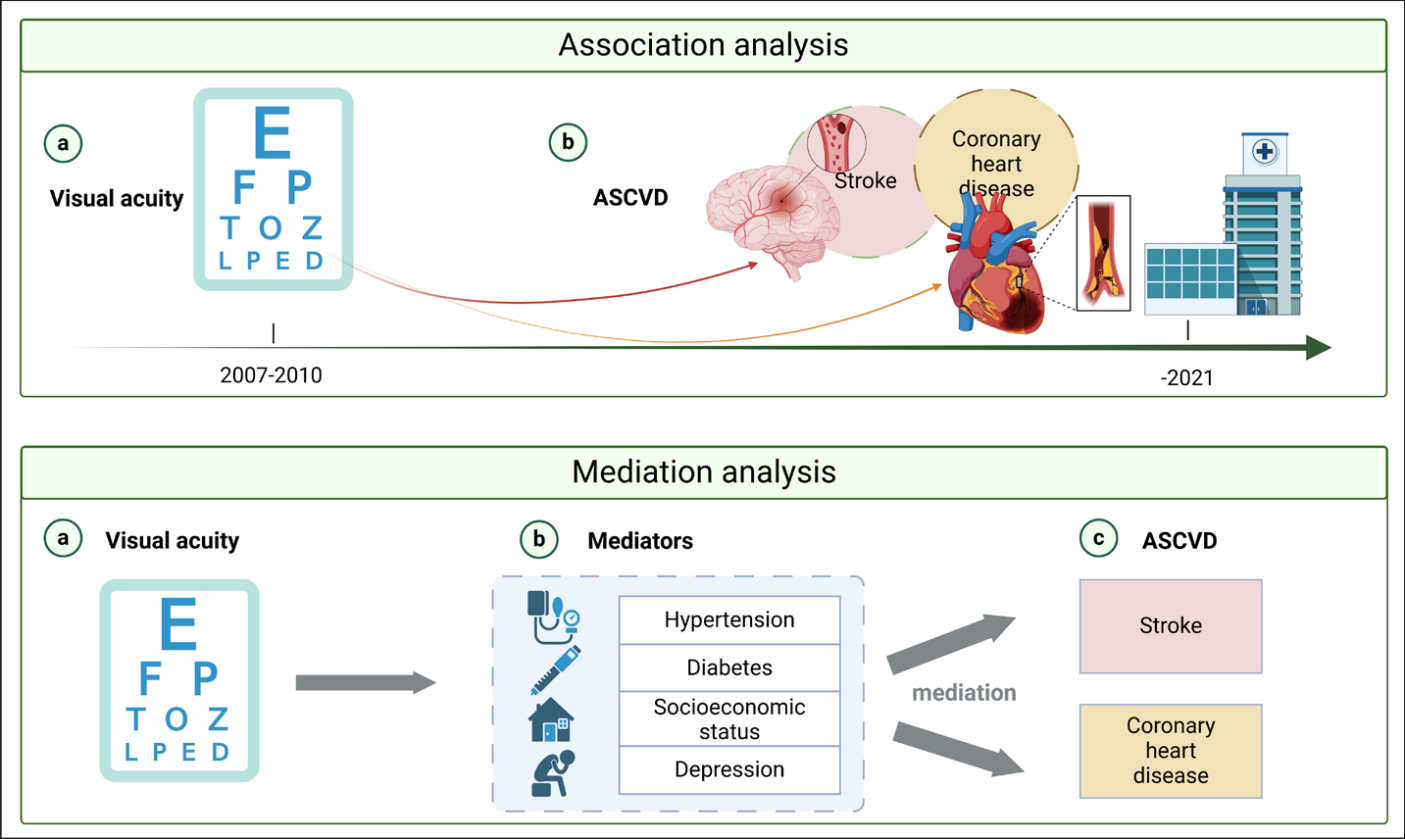
Visual acuity and ASCVD in middle-aged and older adults.

## What is already known

- Visual impairment, including glaucoma and diabetic retinopathy, which affect vision, is associated with risk factors for atherosclerotic cardiovascular disease (ASCVD), such as diabetes and hypertension. However, the association between the full spectrum of visual acuity (VA) and ASCVD has not been fully explored. Nevertheless, there is still a lack of research examining the common risk factors for VA and ASCVD and their mediating effects.

## What this study adds

- This study identifies that visual decline is independently associated with an increased risk of ASCVD, highlighting the importance of early intervention through regular eye exams.
- It reveals that depression and socioeconomic status have a greater mediating effect on the VA-ASCVD association than traditional risk factors like hypertension and diabetes.
- The findings underscore the need to address mental health and socioeconomic disparities to reduce ASCVD risk, particularly in older women with visual impairments.

## Introduction

As the leading cause of morbidity and mortality worldwide, atherosclerotic cardiovascular disease (ASCVD) events account for approximately 17.6 million deaths annually and have significant financial impacts worldwide (1). The recently published ACC/AHA Guidelines on Prevention of Atherosclerotic Cardiovascular Disease in Adults emphasize the usefulness of a risk-based approach to CVD and implementing lifestyle and medical interventions to prevent adverse cardiovascular events (2). Observational studies further demonstrate that while genetics cause a low-level ASCVD risk, much of this can be overcome by maintaining a healthy lifestyle and avoiding risk factors throughout early life (3).

Visual acuity (VA) is associated with adverse physical and psychological outcomes (4). Research shows that visually impaired adults face a higher risk of cardiovascular disease (CVD) and mortality compared to those with normal vision (4,5). This association may be partially attributed to conditions such as glaucoma and diabetic retinopathy, which are strongly linked to traditional ASCVD risk factors like diabetes and hypertension (6,7,8). Moreover, authoritative guidelines emphasize the importance of managing diabetes and hypertension to mitigate ASCVD risk (9,10,11). These two conditions, as major contributors to both cardiovascular risks and retinal complications, highlight their role as shared pathways connecting visual impairment (VI) and cardiovascular health.

Beyond these traditional risk factors, depression and socioeconomic status also play significant roles in both visual and cardiovascular health. Depression has emerged as a major contributor to ASCVD, with evidence pointing to mechanisms such as chronic inflammation, hormonal imbalances, and reduced health behaviors (12,13). Similarly, socioeconomic status influences health outcomes by limiting access to medical care, increasing exposure to chronic stress, and exacerbating health disparities (14,15). Studies suggest that individuals with low socioeconomic status are more likely to experience both ASCVD and poorer vision, possibly due to barriers to healthcare, education, and access to nutritious food (16,17,18). Depression and socioeconomic deprivation have also been associated with delayed eye care and reduced quality of life, further compounding the risk of both VI and ASCVD (19).

Despite these well-established associations, there is limited evidence exploring whether VA itself serves as an independent predictor of ASCVD or whether the relationship is mediated through long-term exposure to these risk factors. Given that a large proportion of ASCVD events occur in patients without known cardiovascular disease (2), exploring the association between VA and ASCVD may help provide a comprehensive framework for risk stratification and early intervention strategies to reduce ASCVD incidence and related mortality.

Building on the well-documented relationships between VA and key risk factors for ASCVD, including hypertension, diabetes, depression, and socioeconomic status, we hypothesized that VA would independently predict ASCVD events. Additionally, given the dual impact of these factors on both visual and cardiovascular health, we proposed that the association between VA and ASCVD may be partly mediated through these pathways. To test these hypotheses, we utilized the UK Biobank, a large and diverse prospective cohort study, enabling a longitudinal analysis of the relationships and pathways between VA and ASCVD.

## Methods

### Study Sample

The UK Biobank is a national, prospective study aimed at identifying determinants of complex diseases and improving health outcomes in British adults. Of 9.2 million NHS participants aged 40–69 years invited, over 500,000 (5.5% response rate) participated in baseline assessments from 2006 to 2010. Data collection included questionnaires, physical measures, and biological samples, with long-term follow-up for health outcomes (20). Ophthalmic assessments were introduced to the baseline assessment in 2009 for six assessment centers.

The UK Biobank received ethical approval (Ref 11/NW/0382), and informed consent was obtained from all participants. Methods, protocols, and definitions are detailed on the UK Biobank website (https://www.ukbiobank.ac.uk/). Data for this analysis were provided under project reference #86091, with the participant selection process outlined in Supplementary Figure 1.

### Visual Acuity Testing at Baseline

The procedure for VA testing in the UK Biobank Study has been described in detail elsewhere (21). Presenting distance, VA was measured with corrections (if any) at a 4 m distance using the logarithm of the minimum angle of resolution (logMAR) chart (Precision Vision, LaSalle, Illinois, USA) on a computer screen. Presenting VA was scored as the total number of correctly read lines, converted to logMAR units, where lower values indicate better visual acuity and higher values indicate poorer visual acuity. The present analysis is based on the presenting VA in the better-seeing eye. VA was modeled as both a continuous variable (per 0.1 logMAR increase) to evaluate the linear association between VA and ASCVD risk and as a categorical variable divided into tertiles (T1: highest VA, T3: lowest VA) to examine potential dose-response relationships.

### Ascertainment of Incident Atherosclerotic Cardiovascular Disease

ASCVD cases in the UK Biobank Study were ascertained by combining data from participants’ medical history (UK Biobank Field 20002), record linkage to hospital admissions data (UK Biobank Field 41270), and the national death register (UK Biobank Field 40001). The hospital admissions data and the national death register data were defined using the International Classification of Diseases (ICD) codes. Incident ASCVD was defined as the first occurrence of coronary heart disease (CHD) events (myocardial infarction (ICD-10 codes I21–I23), resuscitated cardiac arrest (ICD-10 codes I46.0), and fatal CHD (ICD-10 codes I20–I25)) or stroke (fatal and non-fatal events (ICD-10 codes I60–I64)). The present study excluded participants with ASCVD diagnosis at baseline. Follow-up time was calculated as the duration between the date of the first assessment and the earliest occurrence of ASCVD, death, loss to follow-up, or the end of the study period.

### Potentially mediating factors

In this study, the potential mediators for the association between VA and ASCVD that were selected for mediation analysis included hypertension, diabetes, depression, and Townsend deprivation indices. A significant indirect role (mediation) was deemed present when the following conditions were met: (a) a significant relationship existed between the independent variable and the mediator, (b) a significant relationship existed between the independent variable and the dependent variable, (c) a significant relationship existed between the mediator and the dependent variable, and (d) the association between the independent and dependent variables, known as the direct role, was attenuated when the mediator was included in the regression model (22).

For baseline assessment: 1) hypertension was defined as self-reported (UK Biobank field: 20002), the use of antihypertensive drugs (UK Biobank field: 6153), and average systolic blood pressure of at least 130 mmHg (UK Biobank field: 4080) or average diastolic blood pressure of at least 80 mmHg (UK Biobank field: 4079); 2) diabetes was defined as doctor-diagnosed diabetes mellitus, (UK Biobank field: 2443), the use of anti-hyperglycemic medications (UK Biobank field: 20003) or insulin (UK Biobank field: 6153), or glycosylated hemoglobin level of >6.5% (UK biobank field: 30750); 3) depression was defined as self-reported (UK Biobank field: 20002) or had a score on the Patient Health Questionnaire (PHQ, the first two items) of at least 3 (UK Biobank field: 2050 and 2060); 4) each participant was assigned a Townsend deprivation index (UK Biobank field: 189), which is used as a proxy for socioeconomic status and calculated based on the preceding national census output areas. The score is based on four variables: households without a car, overcrowded households, households not owner-occupied, and persons unemployed (23).

For longitudinal assessment, linked hospital admission records also identified a primary or secondary diagnosis of hypertension (ICD-10 codes I10–I15), diabetes (ICD-10 codes E10–E14) and depression (ICD-10 codes F32 and F33). And these cases only occurred after the date of baseline assessment and before the date of onset ASCVD was included in the mediation analysis.

### Covariates

Potential confounders in the present analysis include age, sex, ethnicity (recorded as white and non-white), education attainment (recorded as college or university degree, and others), family history of ASCVD (a marker of biological vulnerability), smoking status (recorded as current/previous and never), physical activity level (recorded as above moderate/vigorous/walking recommendation or not) and hyperlipidemia (defined as self-reported, the use of hyperlipidemia-related medication or statins or with a blood cholesterol level ≥ 6.21 mmol/L), systolic and diastolic blood pressure (measured in mmHg), HbA1c levels (measured in mmol/mol), LDL (measured in mmol/L), HDL (measured in mmol/L), triglycerides (measured in mmol/L), and total cholesterol (measured in mmol/L), all of which were collected at the same time as the VA data. All the variables used in the paper are detailed in Supplementary Table 1 in the data supplement. Baseline measurements of hypertension, diabetes, depression, and Townsend deprivation indices were also considered as covariates in the exploration of the association between VA and ASCVD.

### Statistical Analysis

Data to describe baseline participant information was expressed descriptively, with mean and standard deviation (SD) describing continuous variables and frequency and percentages to describe categorical variables. Baseline characteristics across VA tertiles were compared using ANOVA for continuous variables and Chi-square tests for categorical variables. *P*-values represent overall differences among the three groups.

The association between baseline VA and incident ASCVD was estimated by Cox proportional hazards regression models. The first model adjusted for baseline measurements of age and gender, while the second model additionally included baseline measurements of ethnicity, smoking status, Townsend index, education level, family history of ASCVD, physical activity level, and comorbidities (depression, diabetes, hypertension, and hyperlipidemia). Two sensitivity analyses were conducted to examine the robustness of these results. First, we excluded all incident ASCVD cases that occurred within the first two years of follow-up to minimize the potential for reverse causality. Second, we further adjusted for clinically relevant variables, including systolic and diastolic blood pressure, HbA1c levels, LDL, HDL, and total cholesterol, to ensure that the findings remained consistent under additional levels of adjustment.

We conducted mediation analysis using a four-way decomposition approach to estimate the direct and indirect effects of VA on ASCVD outcomes through potential mediators, including hypertension, diabetes, depression, and socioeconomic status (Townsend index). Specifically, we assessed mediation effects for the overall ASCVD outcome and for its two subcomponents, CHD and stroke, to better understand their unique pathways. Mediation analysis was performed using the ‘med4way’ command in STATA (24). This method decomposes the total effect (TE) into four components: controlled direct effect (CDE, the effect is neither due to the mediator nor exposure-mediator interactions), reference interaction (INTref, the effect is only from interaction), mediated interaction (INTmed, the effect from interaction is only active when mediation is present), and the pure indirect effect (PIE, the effect due to mediation alone). A Weibull distribution fitted the outcome (years of follow-up before incident ASCVD) with an accelerated failure time (AFT) and a logistic regression model for the mediator. The proportion mediated by each mediator was also calculated. To quantify the magnitude of mediation, the study estimated the proportion of the association mediated by mediators (PIE/TE). To address potential confounding, covariates such as age, sex, ethnicity, smoking status, education level, physical activity, and hyperlipidemia were included in the models, as these variables could influence both the exposure (VA) and the outcome (ASCVD). However, the mediator under analysis was not included as a covariate when evaluating its mediation effect to avoid over-adjustment bias and preserve the integrity of the causal pathway. In the primary mediation analysis, other mediators were included as covariates to control for their potential influence on the mediator-outcome relationship and to obtain more precise estimates of the indirect effects. To test the robustness of our findings, a sensitivity analysis was performed in which other mediators were excluded as covariates. For each subcomponent (CHD and stroke), we evaluated the indirect effects of mediators using the same approach as for the overall ASCVD analysis.

Data analyses were conducted using Stata version 16 (StataCorp), and all *P*-values were 2-sided with statistical significance set at <0.05.

## Results

Of the 502,395 UK Biobank participants enrolled between 2006 and 2010, VA was measured in 117,218 (23.33%) participants. After excluding participants diagnosed with ASCVD prior to baseline assessment (n = 6,696), a total of 110,522 participants were included in the final analysis (55.53% females; mean age 56.55 [8.12] years). Table 1 describes baseline characteristics for included participants stratified by VA tertiles. Compared to those in the lowest tertile of VA (the highest visual acuity level), individuals with lower visual acuity were older, more likely to be women, had lower educational qualifications, higher area deprivation, and were more likely to be current smokers. They were also more likely to have a family history of ASCVD and a history of systemic diseases (hypertension, diabetes, hyperlipidemia, or depression). Additionally, individuals in the lowest VA tertile had higher systolic and diastolic blood pressure, HbA1c, HDL, triglyceride, and total cholesterol levels (all *P* < 0.05, representing overall differences across tertiles).

**Table 1:** Cohort characteristics by VA tertile.


CHARACTERISTIC	HIGHEST VA LEVEL (N = 39,168)	MIDDLE VA LEVEL (N = 36,130)	LOWEST VA LEVEL (N = 35,224)	*P* VALUE

**Age, mean (SD), yrs**	54.17 (8.23)	56.94 (7.92)	58.81 (7.45)	**<0.001**

**Gender, No. (%)**				**<0.001**

Women	19,840 (50.65)	20,888 (57.81)	20,772 (58.97)	

Men	19,328 (49.35)	15,242 (42.19)	14,452 (41.03)	

**Ethnicity, No. (%)**				**<0.001**

White	35,442 (90.49)	32,246 (89.25)	30,653 (87.02)	

Non-white	3,726 (9.51)	3,884 (10.75)	4,571 (12.98)	

**Townsend index, mean (SD**)	–1.18 (2.89)	–0.99 (2.98)	–0.69 (3.11)	**0.004**

**Education level, No. (%)**				**<0.001**

College or university degree	15,730 (40.16)	12,665 (35.05)	10,626 (30.18)	

Others	23,438 (59.84)	23,465 (64.95)	24,598 (69.82)	

**Smoking status, No. (%)**				**<0.001**

Never	22,315 (57.24)	20,197 (56.21)	19,378 (55.52)	

Former/current	16,667 (42.76)	15,732 (43.79)	15,527 (44.48)	

**Family history of ASCVD, No. (%)**				**<0.001**

No	18,880 (48.20)	16,242 (44.95)	15,477 (43.94)	

Yes	20,288 (51.80)	19,888 (55.05)	19,747 (56.06)	

**Physical activity, No. (%)**				0.062

Not meeting recommendation	5,576 (17.06)	5,210 (17.75)	4,902 (17.58)	

Meeting recommendation	27,105 (82.94)	24,147 (82.25)	22,980 (82.42)	

**History of diabetes, No. (%)**				**<0.001**

No	37,449 (95.61)	34,054 (94.25)	32,517 (92.31)	

Yes	1,719 (4.39)	2,076 (5.75)	2,707 (7.69)	

**History of hypertension, No. (%)**				**<0.001**

No	11,494 (29.35)	9,430 (26.10)	7,982 (22.66)	

Yes	27,674 (70.65)	26,700 (73.90)	27,242 (77.34)	

**History of hyperlipidemia, No. (%)**				**<0.001**

No	23,500 (60.00)	20,034 (55.45)	18,294 (51.94)	

Yes	15,668 (40.00)	16,096 (44.55)	16,930 (48.06)	

**History of depression, No. (%)**				**<0.001**

No	37,196 (94.97)	34,105 (94.40)	33,184 (94.21)	

Yes	1,972 (5.03)	2,025 (5.60)	2,040 (5.79)	

**Systolic blood pressure**	135.33 (17.72)	137.49 (18.49)	139.18 (18.75)	**<0.001**

**Diastolic blood pressure**	81.81 (9.92)	82.00 (10.02)	82.22 (9.99)	**<0.001**

**HbA1c, mean (SD)**	35.33 (5.71)	36.09 (6.36)	36.89 (7.59)	**<0.001**

**LDL, mean (SD)**	3.57 (0.83)	3.57 (0.85)	3.57 (0.87)	0.91

**HDL, mean (SD)**	1.46 (0.38)	1.49 (0.39)	1.49 (0.40)	**<0.001**

**Triglycerides, mean (SD)**	1.68 (0.99)	1.68 (0.96)	1.70 (0.98)	**0.001**

**Cholesterol, mean (SD)**	5.71 (1.09)	5.74 (1.12)	5.74 (1.15)	**<0.001**

**LogMAR of visual acuity (better eye)**	–0.18 (0.05)	–0.07 (0.26)	0.13 (0.14)	**<0.001**


Abbreviation: ASCVD, atherosclerotic cardiovascular disease; LogMAR, logarithm of the minimum angle of resolution; HbA1c, Glycated Hemoglobin; LDL, Low-Density Lipoprotein; HDL, High-Density Lipoprotein.

### Association of VA and Incident ASCVD

Over a median (interquartile range, IQR) follow-up duration of 11.10 (10.91–11.37) years, a total of 5,496 (4.97%) cases of incident ASCVD were documented. Baseline characteristics stratified by incident ASCVD are described in Supplementary Table 2. The incident ASCVD group was more likely to be older, male, and of non-white ethnicity than the non-ASCVD group. They also had a higher proportion of lower socioeconomic status, education attainment, and physical activity but higher rates of family history of ASCVD, smokers, depressives, diabetics, hypertensives, and hyperlipidemics. Furthermore, the incident ASCVD group had poorer VA than the normal group, especially in women (*P* < 0.001) (Supplementary Figure 2). A significant association between baseline VA and incident ASCVD was observed ([Hazard ratio (HR) = 1.97; 95% confidence interval (95%CI): 1.68–2.31, *P* < 0.001) after adjusting for age and gender. After multivariable adjustment, a one-line worse VA (0.1 logMAR increase) remained significantly associated with a 63% higher risk of incident ASCVD (95%CI: 1.35–1.96, P < 0.001). When dividing logMAR VA into low, moderate, and high tertiles, a significant dose-response relationship was observed between ASCVD risk and worsening VA across groups (*P* for trend <0.001). Similar trends emerged from the sex-stratified analyses (*P* for interaction = 0.867; female: HR = 1.46, 95%CI: 1.06–2.02, *P* = 0.020; male: HR = 1.73, 95%CI: 1.37–2.18, *P* < 0.001) (Table 2). Similar findings were observed after excluding participants diagnosed with ASCVD within the first two years of follow-up and further adjusting for clinically relevant variables (Supplementary Table 3 and Supplementary Table 4).

**Table 2:** Cox Proportional Hazards Models for Incident ASCVD by VA.


	MODEL 1		MODEL 2	
	
	HR (95% CI)	P VALUE	HR (95% CI)	P VALUE

**Total analysis**				

**Visual acuity (Continuous variable, per 0.1 LogMAR)**	1.97 (1.68, 2.31)	<0.001	1.63 (1.35, 1.96)	<0.001

**Visual acuity (Categorical variable)**				

T1 (highest visual acuity level)	1 [Reference]		1 [Reference]	

T2 (moderate visual acuity level)	1.10 (1.03, 1.18)	0.005	1.02 (0.94, 1.10)	0.627

T3 (lowest visual acuity level)	1.30 (1.20, 1.37)	<0.001	1.17 (1.08, 1.26)	<0.001

**P for trend**		<0.001		<0.001

**Stratification analysis**				

**Stratified by gender**				

Female	1.93 (1.49, 2.51)	<0.001	1.46 (1.06, 2.02)	0.020

Male	1.99 (1.62, 2.43)	<0.001	1.73 (1.37, 2.18)	<0.001

**Test for interaction**		0.725		0.771


We used Cox proportional hazards regression for the incident ASCVD. Model 1 was adjusted for baseline measurements of age and gender. Model 2 additionally adjusted for risk factors shared between VA and ASCVD, measured at baseline, including ethnicity, smoking status, education level, Townsend index, family history of severe ASCVD, physical activity level, and comorbidities (depression, diabetes, hypertension, and hyperlipidemia). T, tertiles of LogMAR value. VA, visual acuity; ASCVD, atherosclerotic cardiovascular disease; HR, hazard ratio; LogMAR, logarithm of the minimum angle of resolution.

### Potential mediators of the association between VA and incident ASCVD

**Figure 1:**
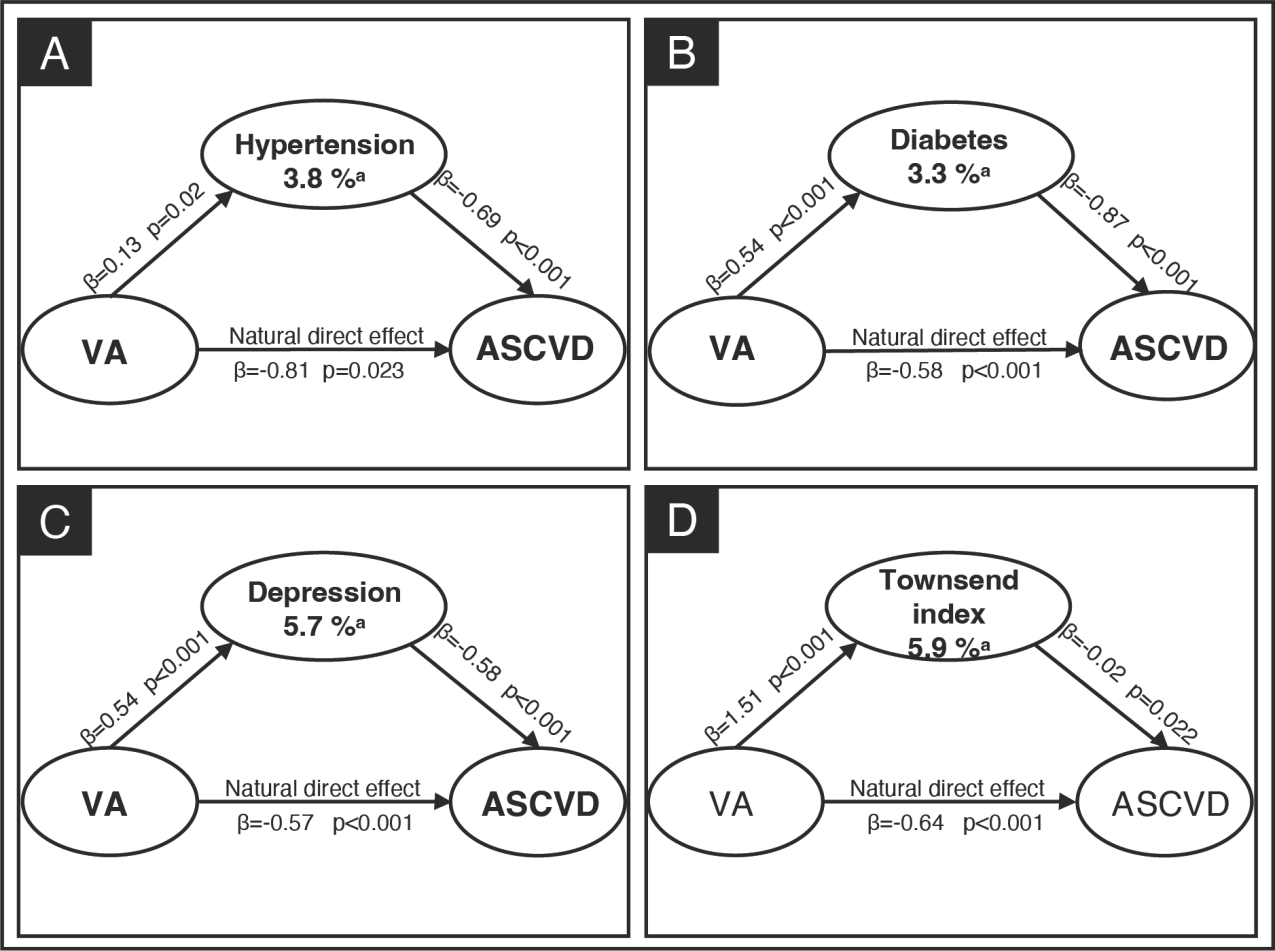
Mediation analysis for VA and ASCVD. Mediation analyses were performed for hypertension (panel **A**), diabetes (panel **B**), depression (panel **C**), and Townsend index (panel **D**) separately. Apart from the mediator being analyzed, other mediators were included as covariates to account for their potential influence on the mediator-outcome relationship. Additionally, the analysis adjusted for baseline measurements of age, gender, ethnicity, smoking status, education level, Townsend index, family history of severe ASCVD, physical activity level, and hyperlipidemia. ^a^P for indirect effect <0.05. VA, visual acuity; ASCVD, atherosclerotic cardiovascular disease.

**Figure 2:**
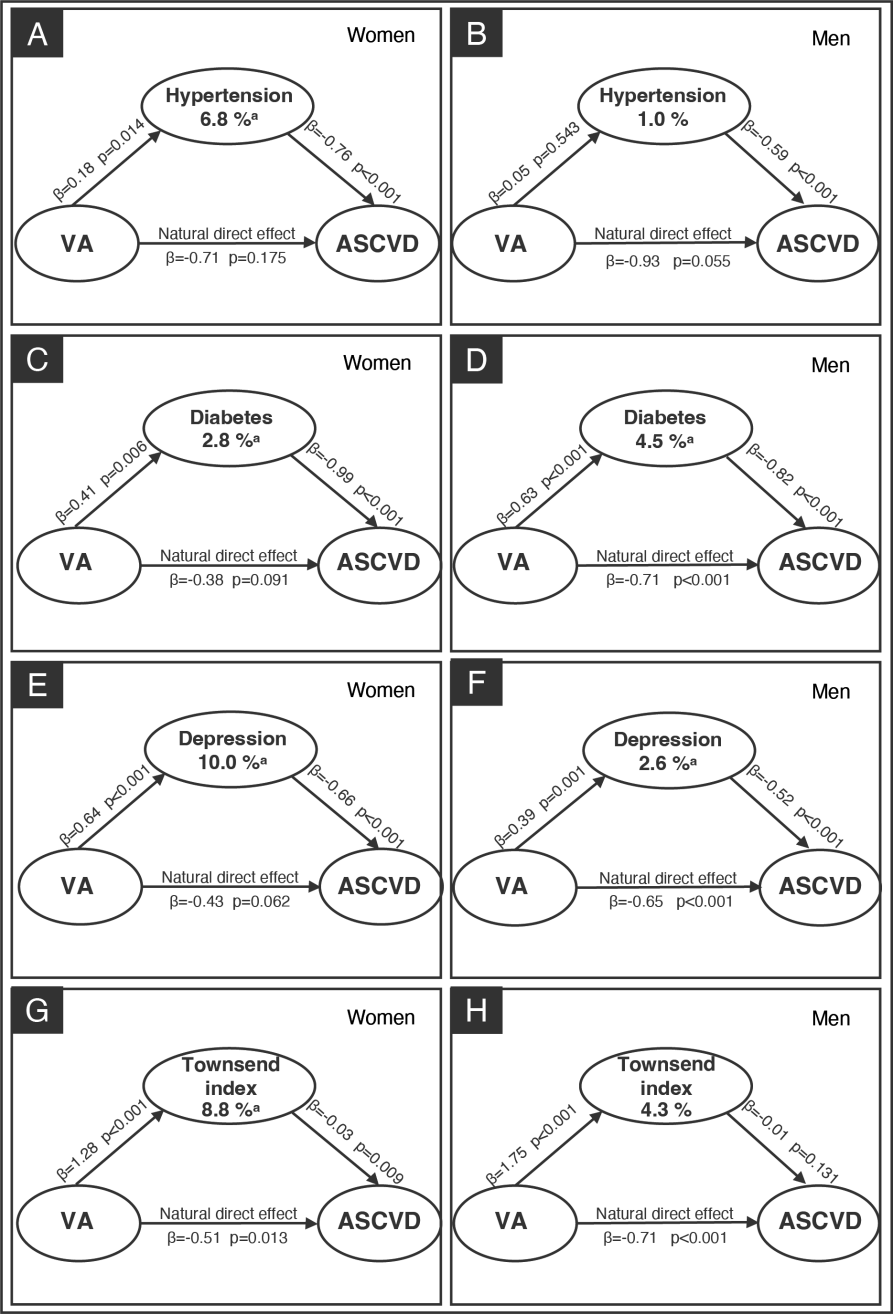
Mediation analysis of sex-specific for VA and ASCVD. For women, mediation analyses were performed for hypertension (panel **A**), diabetes (panel **C**), depression disorder (panel **E**) and Townsend index (panel **G**) separately. For men, mediation analyses were performed for hypertension (panel **B**), diabetes (panel **D**), depression (panel **F**), and Townsend index (panel **H**) separately. Apart from the mediator being analyzed, other mediators were included as covariates to account for their potential influence on the mediator-outcome relationship. Additionally, the analysis adjusted for baseline measurements of age, gender, ethnicity, smoking status, education level, Townsend index, family history of severe ASCVD, physical activity level, and hyperlipidemia. ^a^P for indirect effect <0.05. VA, visual acuity; ASCVD, atherosclerotic cardiovascular disease.

A mediation analysis assessed whether the association between VA and incident ASCVD was mediated through hypertension, diabetes, depression, and the Townsend deprivation index (Figure 1). In this analysis, hypertension (panel A), diabetes (panel B), depression (panel C), and Townsend index (panel D) explained 3.8%, 3.3%, 5.7%, and 5.9% of the estimated effects of VA on incident ASCVD, respectively (all *P* for indirect effect <0.05), after adjusting for all confounders. We repeated the mediation analyses stratified by sex and found that among women, hypertension, diabetes, depression, and Townsend index mediated 6.8%, 2.8%, 10.0%, and 8.8% of the indirect association between VA and ASCVD, respectively (all *P* for indirect effect <0.05). Among men, diabetes and depression made up 4.5% and 2.6% of the association between VA and ASCVD, respectively (all *P* for indirect effect <0.05). Hypertension and Townsend deprivation index were not significant mediators among men (Figure 2). Further details on the mediation analysis are provided in Supplementary Table 5. The results of sensitive analyses remained consistent with the primary analysis, demonstrating that the indirect effects of the mediators were not significantly influenced by the inclusion or exclusion of additional mediators (Supplementary Table 6). An additional analysis evaluated the mediating effect of VA on the incidence of ASCVD by event type (CHD and stroke). The magnitude of the mediation effect was highest in the VA-Stroke group, with hypertension, diabetes, depression, and the Townsend deprivation index explaining 27.9%, 4.3%, 6.9%, and 15.7% of the association, respectively, compared to the VA-CHD group (3.8%, 3.6%, 6.0%, and 7.0%, respectively) (all *P* for indirect effect <0.05) (Supplementary Figure 3).

## Discussion

In a large population of middle-aged and older adults, our study demonstrates that visual decline is associated with incident ASCVD, with a clear trend observed between the severity of visual decline and the risk of incident ASCVD. Furthermore, we provide evidence that several shared risk factors for both VA and ASCVD likely mediate this association. In particular, hypertension, diabetes, depression, and Townsend deprivation index influenced 3.8%, 3.3%, 5.7%, and 5.9% of this association, respectively. When analyzed by gender subgroups, the largest indirect association was observed for depression (10.0%) among women and diabetes (4.5%) among men.

Our findings suggest that declines in VA confer an increased risk for incident cardiovascular diseases. This is consistent with Thiagarajan et al. (25), who discovered in UK subjects 75 years and older that VI without ocular disease was associated with a small but significant increase in all-cause mortality and cardiovascular mortality. Another large cohort study of young and middle-aged Koreans demonstrated that VI was significantly associated with an increased risk of all-cause, injury-related, and cardiovascular mortality (26). Moreover, Lee et al. (27) showed in an American cohort that women, but not men, with severe bilateral self-reported VI were at significantly increased risk of cardiovascular mortality compared to those with no VI in fully adjusted models. While VI has been the subject of many studies, none so far have assessed the full spectrum of VA and incident ASCVD like the current research. Our analysis shows a positive independent association was pronounced in women and men across the VA spectrum. Therefore, visual decline should alert practitioners to potential vascular dysfunction and present an opportunity for the assessment of ASCVD risk factors.

Our analysis provides a better understanding of the mechanisms connecting VA to ASCVD, specifically hypertension, diabetes, depression, and Townsend deprivation index, which were identified as indirect mediators in the observed associations. Although these are well-known risk factors for both VA and ASVD, this is the first study to document that they mediate the precipitation of adverse health events through an association with VA. Ocular complications of systemic diseases, such as glaucoma associated with hypertension and diabetic retinopathy related to diabetes, are known to be indicators of longstanding risk factors for ASCVD and cause irreversible VI (28). More surprisingly, depression and socioeconomic status accounted for much higher mediatory effects than hypertension and diabetes in the VA-ASCVD relationship, even though hypertension and diabetes are traditionally among the highest risk factors for ASCVD events (29,30). In fact, clinical practice guidelines suggest people living with mental health problems should be considered disadvantaged from their higher CVD risk (31). This is particularly relevant for individuals with VI, who are at a higher risk of developing depression due to reduced quality of life, social isolation, and decreased physical activity, further compounding their vulnerability to adverse cardiovascular outcomes (32,33,34). Incorporating mental health assessments and interventions into routine care for visually impaired individuals could play a pivotal role in reducing the compounding effects of mental health issues on vascular health. Socioeconomic status significantly limits access to healthcare resources, which increases ASCVD risk through mechanisms such as long-term uncorrected VI, delayed diagnosis and treatment, and poorer vision-targeted health-related quality of life (35,36). Studies have shown that individuals with lower socioeconomic status are less likely to receive timely and adequate eye care, exacerbating preventable VI and its associated health risks (37). Socioeconomic inequalities significantly contribute to disparities in cardiovascular outcomes, including higher rates of ASCVD events among lower-income groups (38). This highlights the need for equitable healthcare systems to ensure socioeconomic status is not a barrier to quality medical and eye care, reducing health disparities. Traditional risk factors like hypertension and diabetes, while important, may not fully capture the risk profile for ASCVD in populations with VI. Integrating mental health and socioeconomic assessments into routine clinical practice could provide a more holistic approach to managing and mitigating ASCVD risk in visually impaired individuals.

Mediators of the VA-ASCVD association varied greatly across genders, with women appearing to bear a disproportionately higher burden of VA-ASCVD risk. The most prominent mediator in women was depression, accounting for 10.0% of the mediation effect, compared to diabetes in men, which accounted for 4.5%. Women are nearly twice as likely to suffer from an episode of depression as men (39,40), and the association between depression duration and incident CVD is particularly strong in women (41). In contrast, men have a 61% higher likelihood of developing diabetes than women (42,43), which may explain its stronger mediatory effect in men. Besides, the socioeconomic effect in this study also biased women (8.8%) more than men. A previous study indicated that women from low-income households had higher risk status, greater risk factor burden, and more prevalent coronary disease (44). Our findings suggest that women with lower vision are the vulnerable populations for disparities in ASCVD through a socioeconomic status mediation pathway. An integrated health approach addressing the impacts of socioeconomic status and gender on visual and cardiovascular health is vital for reducing disparities and improving the well-being of women with visual impairments.

This study highlights that mediators for VA-ASCVD associations were markedly different when analyzed separately for CHD and stroke. The VA-stroke association was profoundly influenced by indirect associations, notably hypertension (27.9%) and Townsend deprivation index (15.7%). Hypertension’s role as a key mediator aligns with a pooled analysis showing that blood pressure is the most significant risk factor for stroke, accounting for over two-thirds of the total risk (46). As this is the first study to examine the dominating effects of mediators on VA-ASCVD and its subcomponents, our findings emphasize the substantial risk of VA-stroke mediated by hypertension and socioeconomic status. These results highlight the need for targeted interventions, such as enhanced blood pressure control and policies addressing socioeconomic disparities, to mitigate stroke risk in populations with visual impairment.

Our study was based on a prospective, large cohort with long-term follow-up that followed a standardized protocol for ocular and in-person assessments. Moreover, the UK Biobank routinely updates health-related records to identify incident ASCVD, which allowed us to comprehensively and longitudinally examine the association between visual health and ASCVD and their mediators. These features strengthen the reliability of the data and the integrity of our findings; however, some limitations should be acknowledged. First, we cannot exclude selection and collider bias, as there were differences in the frequencies of the outcome (incident ASCVD), exposure (VA), and mediators (hypertension, diabetes status, depression, and Townsend deprivation index) of interest that were not eligible for the current analysis (47). Nevertheless, selection bias is unlikely to distort our results considerably, given our findings followed a dose-response relationship. Secondly, the UK Biobank is representative of a largely white first-world country, which inhibits these findings from being generalized to multi-ethnic populations and countries of dissimilar demographic status. In consideration, it would be ideal for these findings to be examined across ethnicities and geographic regions with marked differences.

## Conclusion

In summary, our findings suggest the visual decline is associated with ASCVD independently, with hypertension, diabetes, depression and socioeconomic status partly explaining the association between VA and incident ASCVD in the UK Biobank cohort. Depression and socioeconomic status had a stronger mediation effect than traditional factors like hypertension and diabetes. Taken together with a growing body of literature, our results suggest clinicians caring for adults with ASCVD and poorer vision should be aware depression and socioeconomic status are important mediators of ASCVD event risk, particularly in women.

## Data Accessibility Statement

This project corresponds to UK Biobank application ID#86091. Data from the UK Biobank dataset are available at https://biobank.ndph.ox.ac.uk/ by application.

## Additional File

The additional file for this article can be found as follows:

10.5334/gh.1406.s1 Supplementary Online Content. Supplemental data include 6 tables and 3 figures.

## Abbreviations

VA, visual acuity; ASCVD, atherosclerotic cardiovascular disease; CI, confidence interval; HR, hazard ratios; ICD-10, the 10th edition of the WHO International Classification of Diseases; LogMAR, logarithm of the minimum angle of resolution; MHQ, mental health questionnaire; OR, odds ratio; SD, standard deviation.

## Code Availability

STATA command *med4way* was used to perform causal mediation analysis with survival data (https://raw.githubusercontent.com/anddis/med4way/master/).

## References

[1] Virani SS, Alonso A, Benjamin EJ, Bittencourt MS, Callaway CW, Carson AP, et al. Heart disease and stroke statistics—2020 update: A report from the American Heart Association. Circulation. 2020;141(9):e139–e596. 31992061 10.1161/CIR.0000000000000757

[2] Goff DC Jr., Lloyd-Jones DM, Bennett G, Coady S, D’Agostino RB, Gibbons R, et al. 2013 ACC/AHA guideline on the assessment of cardiovascular risk: A report of the American College of Cardiology/American Heart Association Task Force on Practice Guidelines. Circulation. 2014;129(25_suppl_2):S49–S73. DOI: 10.1161/01.cir.0000437741.48606.98 24222018

[3] Berry JD, Dyer A, Cai X, Garside DB, Ning H, Thomas A, et al. Lifetime risks of cardiovascular disease. New England Journal of Medicine. 2012;366(4):321–329. DOI: 10.1056/NEJMoa1012848 22276822 PMC3336876

[4] Christ SL, Lee DJ, Lam BL, Zheng DD, Arheart KL. Assessment of the effect of visual impairment on mortality through multiple health pathways: structural equation modeling. Investigative Ophthalmology & Visual Science. 2008;49(8):3318–3323. DOI: 10.1167/iovs.08-1676 18362104

[5] Rajala U, Pajunpää H, Koskela P, Keinänen-Kiukaanniemi S. High cardiovascular disease mortality in subjects with visual impairment caused by diabetic retinopathy. Diabetes Care. 2000;23(7):957–961. DOI: 10.2337/diacare.23.7.957 10895846

[6] Cheung N, Wong TY. Diabetic retinopathy and systemic vascular complications. Progress in Retinal and Eye Research. 2008;27(2):161–176. DOI: 10.1016/j.preteyeres.2007.12.001 18249026

[7] Ahn SJ, Woo SJ, Park KH. Retinal and choroidal changes with severe hypertension and their association with visual outcome. Investigative Ophthalmology & Visual Science. 2014;55(12):7775–7785. DOI: 10.1167/iovs.14-14915 25395485

[8] Dupas B, Minvielle W, Bonnin S, Couturier A, Erginay A, Massin P, et al. Association between vessel density and visual acuity in patients with diabetic retinopathy and poorly controlled type 1 diabetes. JAMA Ophthalmology. 2018;136(7):721–728. DOI: 10.1001/jamaophthalmol.2018.1319 29800967 PMC6136049

[9] Arnett DK, Blumenthal RS, Albert MA, et al. 2019 ACC/AHA Guideline on the primary prevention of cardiovascular disease: Executive summary: A report of the American College of Cardiology/American Heart Association Task Force on Clinical Practice Guidelines. Circulation. 2019;140(11):e563–e595. DOI: 10.1161/CIR.0000000000000677 30879339 PMC8351755

[10] Cheung BM, Cheng CH, Lau CP, Wong CK, Ma RC, Chu DW, et al. 2016 consensus statement on prevention of atherosclerotic cardiovascular disease in the Hong Kong population. Hong Kong Medical Journal. 2017;23(2):191–201. DOI: 10.12809/hkmj165045 28387202

[11] Rosenblit PD. Extreme atherosclerotic cardiovascular disease (ASCVD) risk recognition. Current Diabetes Reports. 2019;19(8):61. DOI: 10.1007/s11892-019-1178-6 31332544

[12] Kim H, Lee YB, Lee J, Kang D, Kim G, Jin SM, et al. Association between depression, antidepressant use, and the incidence of atherosclerotic cardiovascular diseases. Journal of Affective Disorders. 2024;352:214–221. DOI: 10.1016/j.jad.2024.02.034 38378089

[13] Barger SD, Struve GC. Association of depression with 10-year and lifetime cardiovascular disease risk among US adults, national health and nutrition examination survey, 2005–2018. Preventing Chronic Disease. 2022;19:E28. DOI: 10.5888/pcd19.210418 35617679 PMC9165473

[14] Li R, Hou J, Tu R, Liu X, Zuo T, Dong X, et al. Associations of mixture of air pollutants with estimated 10-year atherosclerotic cardiovascular disease risk modified by socio-economic status: The Henan Rural Cohort Study. The Science of the Total Environment. 2021;793:148542. DOI: 10.1016/j.scitotenv.2021.148542 34174609

[15] Vallée A. Association between socio-economic status and estimated atherosclerotic cardiovascular disease risk: results from a middle-aged population-based study. Public Health.;221:1–9. DOI: 10.1016/j.puhe.2023.05.014 37331308

[16] Hahm BJ, Shin YW, Shim EJ, Jeon HJ, Seo JM, Chung H, et al. Depression and the vision-related quality of life in patients with retinitis pigmentosa. British Journal of Ophthalmology. 2008;92(5):650–654. DOI: 10.1136/bjo.2007.127092 18356260

[17] Varma R, Wu J, Chong K, Azen SP, Hays RD, Group LALES. Impact of severity and bilaterality of visual impairment on health-related quality of life. Ophthalmology. 2006;113(10):1846–1853. DOI: 10.1016/j.ophtha.2006.04.028 16889831

[18] Honigberg MC, Ye Y, Dattilo L, Sarma AA, Scott NS, Smoller JW, et al. Low depression frequency is associated with decreased risk of cardiometabolic disease. Nature Cardiovascular Research. 2022;1(2):125–131. DOI: 10.1038/s44161-021-00011-7 PMC9389944 35991864

[19] Méjean C, Droomers M, Van Der Schouw YT, Sluijs I, Czernichow S, Grobbee DE, et al. The contribution of diet and lifestyle to socioeconomic inequalities in cardiovascular morbidity and mortality. International Journal of Cardiology. 2013;168(6):5190–5195. DOI: 10.1016/j.ijcard.2013.07.188 23998549

[20] Sudlow C, Gallacher J, Allen N, Beral V, Burton P, Danesh J, et al. UK Biobank: An open access resource for identifying the causes of a wide range of complex diseases of middle and old age. PLoS Medicine. 2015;12(3):e1001779. DOI: 10.1371/journal.pmed.1001779 25826379 PMC4380465

[21] Chua SYL, Thomas D, Allen N, Lotery A, Desai P, Patel P, et al. Cohort profile: Design and methods in the eye and vision consortium of UK Biobank. BMJ Open. 2019;9(2):e025077. DOI: 10.1136/bmjopen-2018-025077 PMC6398663 30796124

[22] Zhang X, Wang S, Du Z, Seth I, Wang Y, Liang Y, et al. The associations and mediators between visual disabilities and anxiety disorders in middle-aged and older adults: A population-based study. The American Psychologists. 2023;78(8):982–994. DOI: 10.1037/amp0001143 36848049

[23] Townsend P. Deprivation. Journal of Social Policy. 1987;16(2):125–146. DOI: 10.1017/S0047279400020341

[24] Discacciati A, Bellavia A, Lee JJ, Mazumdar M, Valeri L. Med4way: A Stata command to investigate mediating and interactive mechanisms using the four-way effect decomposition. International Journal of Epidemiology. 2018;48(1)15–20. DOI: 10.1093/ije/dyy236 30452641

[25] Thiagarajan M, Evans JR, Smeeth L, Wormald RP, Fletcher AE. Cause-specific visual impairment and mortality: Results from a population-based study of older people in the United Kingdom. Archives of Ophthalmology. 2005;123(10):1397–1403. DOI: 10.1001/archopht.123.10.1397 16219731

[26] Han SY, Chang Y, Shin H, Choi CY, Ryu S. Visual acuity and risk of overall, injury-related, and cardiovascular mortality: The Kangbuk Samsung Health Study. European Journal of Preventive Cardiology. 2022;29(6):904–912. DOI: 10.1093/eurjpc/zwab025 33615358

[27] Lee DJ, Gómez-Marín O, Lam BL, Zheng DD. Visual acuity impairment and mortality in US adults. Archives of Ophthalmology. 2002;120(11):1544–1550. DOI: 10.1001/archopht.120.11.1544 12427070

[28] Freeman EE, Egleston BL, West SK, Bandeen-Roche K, Rubin G. Visual acuity change and mortality in older adults. Investigative Ophthalmology & Visual Science. 2005;46(11):4040–4045. DOI: 10.1167/iovs.05-0687 16249478

[29] Einarson TR, Acs A, Ludwig C, Panton UH. Prevalence of cardiovascular disease in type 2 diabetes: A systematic literature review of scientific evidence from across the world in 2007–2017. Cardiovascular Diabetology. 2018;17:1–19. DOI: 10.1186/s12933-018-0728-6 29884191 PMC5994068

[30] Whelton PK, Carey RM, Aronow WS, Casey, DE Jr., Collins KJ, Himmelfarb CD, et al. 2017 ACC/AHA/AAPA/ABC/ACPM/AGS/APhA/ASH/ASPC/NMA/PCNA guideline for the prevention, detection, evaluation, and management of high blood pressure in adults: A report of the American College of Cardiology/American heart Association Task Force on Clinical Practice Guidelines. Hypertension. 2018:71(6):1269–1324. DOI: 10.1161/HYP.0000000000000076 29133354

[31] Harshfield EL, Pennells L, Schwartz JE, Willeit P, Kaptoge S, Bell S, et al. Association between depressive symptoms and incident cardiovascular diseases. JAMA. 2020;324(23):2396–2405. DOI: 10.1001/jama.2020.23068 33320224 PMC7739139

[32] Brody BL, Gamst AC, Williams RA, Smith AR, Lau PW, Dolnak D, et al. Depression, visual acuity, comorbidity, and disability associated with age-related macular degeneration. Ophthalmology. 2001;108(10):1893–1900. DOI: 10.1016/S0161-6420(01)00754-0 11581068

[33] Du Z, Zhang X, Hu Y, Huang Y, Bulloch G, Shang X, et al. Association of hyperopia with incident clinically significant depression: Epidemiological and genetic evidence in the middle-aged and older population. The British Journal of Ophthalmology. 2023;107(12):1907–1913. DOI: 10.1136/bjo-2022-321876 36241375 PMC10715478

[34] Du Z, Wang S, Bulloch G, Zhang F, Wang Y, Lai C, et al. Accelerometer-measured daily behaviors that mediate the association between refractive status and depressive disorders. Translational Vision Science & Technology. 2024;13(7):3. DOI: 10.1167/tvst.13.7.3 PMC11221614 38953853

[35] Whillans J, Nazroo J. Social inequality and visual impairment in older people. The Journals of Gerontology, Series B, Psychological Sciences and Social Sciences. 2018;73(3):532–542. DOI: 10.1093/geronb/gbv163 26843396

[36] Owsley C, McGwin G, Scilley K, Meek GC, Seker D, Dyer AJAoO. Effect of refractive error correction on health-related quality of life and depression in older nursing home residents. Archives of Ophthalmology. 2007;125(11):1471–1477. DOI: 10.1001/archopht.125.11.1471 17998508

[37] Besagar S, Yonekawa Y, Sridhar J, Finn A, Padovani-Claudio DA, Sternberg P Jr., et al. Association of socioeconomic, demographic, and health care access disparities with severe visual impairment in the US. JAMA Ophthalmology. 2022;140(12):1219–1226. DOI: 10.1001/jamaophthalmol.2022.4566 36326732 PMC9634598

[38] Yip JL, Luben R, Hayat S, Khawaja AP, Broadway DC, Wareham N, et al. Area deprivation, individual socioeconomic status and low vision in the EPIC-Norfolk Eye Study. Journal of Epidemiology and Community Health. 2014;68(3):204–210. DOI: 10.1136/jech-2013-203265 24179053 PMC4157999

[39] Sassarini J. Depression in midlife women. Maturitas. 2016;94:149–154. DOI: 10.1016/j.maturitas.2016.09.004 27823736

[40] Soares CN. Mood disorders in midlife women: understanding the critical window and its clinical implications. Menopause. 2014;21(2):198–206. DOI: 10.1097/GME.0000000000000193 24448106

[41] Zhang Y, Li X, Chan VK, Luo H, Chan SSM, Wong GHY, et al. Depression duration and risk of incident cardiovascular disease: A population-based six-year cohort study. Journal of Affective Disorders. 2022;305:188–195. DOI: 10.1016/j.jad.2022.03.005 35283180

[42] Aregbesola A, Voutilainen S, Virtanen JK, Mursu J, Tuomainen T-P. Gender difference in type 2 diabetes and the role of body iron stores. Annals of Clinical Biochemistry. 2017;54(1):113–120. DOI: 10.1177/0004563216646397 27166309

[43] Flegal KM, Carroll MD, Ogden CL, Curtin LR. Prevalence and trends in obesity among US adults, 1999–2008. JAMA. 2010;303(3):235–241. DOI: 10.1001/jama.2009.2014 20071471

[44] Shaw LJ, Bairey Merz CN, Bittner V, Kip K, Johnson BD, Reis SE, et al. Importance of socioeconomic status as a predictor of cardiovascular outcome and costs of care in women with suspected myocardial ischemia. Results from the National Institutes of Health, National Heart, Lung and Blood Institute-sponsored Women’s Ischemia Syndrome Evaluation (WISE). Journal of Women’s Health. 2008;17(7):1081–1092. DOI: 10.1089/jwh.2007.0596 PMC2818766 18774893

