# Supplementary material for: Association Between Visual Acuity and Incident Atherosclerotic Cardiovascular Disease: A Longitudinal Test of Mediators
Source: Glob Heart. 2025 Feb 24;20(1):19. doi: 10.5334/gh.1406 (PMC11869825; doi:10.5334/gh.1406)
Supplement: Supplementary Online Content. — Supplemental data include 6 tables and 3 figures. [file gh-20-1-1406-s1.pdf]

Supplementary Online Content

Association between Visual Acuity and Incident Atherosclerotic Cardiovascular Disease: A Longitudinal test of Mediators

Supplemental data include 6 tables and 3 figures.

Supplementary Table 1. UK Biobank Showcase Variables Used in the Study.

Supplementary Table 2. Baseline Characteristics Stratified by Incident ASCVD.

Supplementary Table 3. Sensitivity Analysis: Cox Proportional Hazards Models for Incident ASCVD by VA.

Supplementary Table 4. Sensitivity Analysis: Cox Proportional Hazards Models for Incident ASCVD by VA.

Supplementary Table 5. Mediation Analysis and Four-Way Decomposition of Associations of VA with ASCVD via Potential Mediators.

Supplementary Table 6. Sensitivity Analysis: Mediation Analysis and Four-Way Decomposition of Associations of VA with ASCVD via Potential Mediators.

Supplementary Figure 1. Flow Chart of the Study Sample Selection from the UK Biobank.

Supplementary Figure 2. Mean LogMAR Visual Acuity Stratified by Incident ASCVD.

Supplementary Figure 3. Mediation Analysis for VA, CHD, and Stroke.

This supplementary material has been provided by the authors to give readers additional information about their work.

Supplementary Table 1. UK Biobank Showcase Variables Used in the Study.

Measure	Field ID	Time	Description
**Visual acuity**			
Visual acuity (right), logMAR	5201	Baseline assessment	The procedure for Visual Acuity testing at an Assessment Centre of the UK Biobank follow the manual:https://biobank.ndph.ox.ac.uk/showcase/ukb/docs/Visualacuity.pdf.
Visual acuity (left), logMAR	5208	Baseline assessment	The procedure for Visual Acuity testing at an Assessment Centre of the UK Biobank follow the manual:https://biobank.ndph.ox.ac.uk/showcase/ukb/docs/Visualacuity.pdf.
**Incident ASCVD**			
**Incident ASCVD**	41270 (code I21-I23, I460, I20-25, I60-I64)	From the date of baseline assessment to either the date of onset ASCVD, the date of death, or the end of follow-up (28 April 2021)	Hospital in-patient records with ASCVD as main or any secondary diagnoses based on the 10th edition of the WHO International Classification of Diseases (ICD-10).
	41280		The date each ICD-10 diagnosis code was first recorded in either the primary or secondary position in the participant's hospital inpatient records.
	40001 (code I21-I23, I460, I20-25, I60-I64)		Underlying/primary cause of death reported for participant.
**Mediators**			
Hypertension	20002 (code 1065, 1072)	Baseline assessment	Self-reported hypertension.
	6153 (code 2)		Use of antihypertensive drugs.
	4080		Average systolic blood pressure of at least 130mmHg.
	4079		Average diastolic blood pressure of at least 80mmHg.
Diabetes mellitus	2443	Baseline assessment	Doctor-diagnosed diabetes mellitus. Touchscreen question "Has a doctor ever told you that you have diabetes?"
	20003		The use of anti-hyperglycemic medications.
	6153 (code 3)		The use of insulin.
	30750		Glycated hemoglobin level measured by HPLC analysis on a Bio-Rad VARIANT II Turbo (≥ 48 mmol/mol).
Depression	20002	Baseline assessment	Self-reported depression.
	2050		Frequency of depressed mood in last 2 weeks
	2060		Frequency of unenthusiasm / disinterest in last 2 weeks

Incident hypertension	41270 (code I10-I15)	From the date of baseline assessment to the date of onset anxiety /the date of online mental health questionnaire	Hospital in-patient records with hypertension as main or any secondary diagnosis based on the 10th edition of the WHO International Classification of Diseases (ICD-10).
Incident diabetes	41270 (code E10-E14)		Hospital in-patient records with diabetes as main or any secondary diagnoses based on the 10th edition of the WHO International Classification of Diseases (ICD-10).
Incident depression	41270 (code F32, F33)		Hospital in-patient records with depression as main or any secondary diagnoses based on the 10th edition of the WHO International Classification of Diseases (ICD-10).
Townsend deprivation index	189	Baseline assessment	Townsend deprivation index calculated immediately prior to participant joining UK Biobank based on the preceding national census output areas. Each participant is assigned a score corresponding to the output area in which their postcode is located.
**Demographic information**			
Age	21003	Baseline assessment	Refer to the age of the participant on the day they attended an Assessment Centre, year.
Sex	31	Baseline assessment	Sex of participant.
Ethnic background	21000	Baseline assessment	Recorded as white and non-white.
Family history of ASCVD	20107 (code 1,2)	Baseline assessment	Illnesses of father. Touchscreen question "Has/did your father ever suffer from? (You can select more than one answer)".
	20110 (code 1,2)		Illnesses of mother. Touchscreen question "Has/did your mother ever suffer from? (You can select more than one answer)".
	20111 (code 1,2)		Illnesses of siblings. Touchscreen question "Have any of your brothers or sisters suffered from any of the following diseases? (You can select more than one answer)".
Education attainment	6138	Baseline assessment	Touchscreen question "Which of the following qualifications do you have? (You can select more than one)".
Physical activity levels	22036	Baseline assessment	Indicates whether a person met the 2017 UK Physical activity guidelines of 150 minutes of walking or moderate activity per week or 75 minutes of vigorous activity.
Smoking status	20116	Baseline assessment	This field summarises the current/past smoking status of the participant.
Hyperlipidemia	20002 (code 1473)	Baseline assessment	Self-reported hyperlipidemia.
	6153		The use of statins.
	20003		The use of hyperlipidemia-related medication.
	30690		Blood cholesterol level Measured by CHO-POD analysis on a Beckman Coulter AU5800(≥ 6.21 mmol/L).
Systolic blood pressure	4080	Baseline assessment	Blood pressure, automated reading, systolic. Two measures of blood pressure were taken a few moments apart.
diastolic blood pressure	4079	Baseline	Blood pressure, automated reading, diastolic. Two

		assessment	measures of blood pressure were taken a few moments apart.
HbA1c	30750	Baseline assessment	Measured by HPLC analysis on a Bio-Rad VARIANT II Turbo
LDL	30780	Baseline assessment	Measured by enzymatic protective selection analysis on a Beckman Coulter AU5800
HDL	30760	Baseline assessment	Measured by enzyme immunoinhibition analysis on a Beckman Coulter AU5800
cholesterol	30690	Baseline assessment	Measured by CHO-POD analysis on a Beckman Coulter AU5800
triglycerides	30870	Baseline assessment	Measured by GPO-POD analysis on a Beckman Coulter AU5800

ASCVD, atherosclerotic cardiovascular disease; LogMAR, logarithm of the minimum angle of resolution; HbA1c, Glycated Hemoglobin; LDL, low-density lipoprotein； HDL， high-density lipoprotein.

Supplementary Table 2. Baseline Characteristics Stratified by Incident ASCVD[a].

Baseline Characteristics	Total	Sample without Incident ASCVD	Sample with Incident ASCVD	HR (95% CI)[a]	*P* value
**N**	110,522	105,026 (95.03)	5,496 (4.97)	-	-
**Age, mean (SD), yrs**	56.55 (8.12)	56.33 (8.13)	60.79 (6.60)	**1.08 (1.08, 1.08)**	**<0.001**
**Gender, No. (%)**					
Female	61,500 (55.65)	59,368 (56.53)	2,132 (38.79)	1 [Reference]	
Male	49,022 (44.35)	45,658 (43.47)	3,364 (61.21)	**1.98 (1.83, 2.04)**	**<0.001**
**Ethnicity, No. (%)**					
White	98,341 (88.98)	93,443 (88.97)	4,898 (89.12)	1 [Reference]	
Non-white	12,181 (11.02)	11,583 (11.03)	598 (10.88)	**1.32 (1.21, 1.43)**	**<0.001**
**Townsend index, mean (SD)**	-0.96 (3.00)	- 0.97 (3.00)	- 0.85 (3.06)	**1.03 (1.03, 1.05)**	**<0.001**
**Education level, No. (%)**					
College or university degree	39,021 (35.31)	37,510 (35.71)	1,511 (27.49)	1 [Reference]	
Others	71,501 (64.69)	67,516 (64.29)	3,985 (72.51)	**1.32 (1.25, 1.40)**	**<0.001**
**Smoking status, No. (%)**					
Never	61,890 (56.36)	59,317 (56.84)	2,573 (47.22)	1 [Reference]	
Former/current	47,926 (43.64)	45,050 (43.16)	2,876 (52.78)	**1.25 (1.18, 1.32)**	**<0.001**
**Family history of ASCVD, No. (%)**					
No	50,599 (45.78)	48,750 (46.32)	1,942 (35.33)	1 [Reference]	
Yes	59,923 (54.22)	56,500 (53.68)	3,554 (64.67)	**1.42 (1.34, 1.50)**	**<0.001**
**Physical activity, No. (%)**					
Not meeting recommendation	15,688 (17.45)	14,797 (17.29)	891 (20.55)	1 [Reference]	
Meeting recommendation	74,232 (82.55)	70,787 (82.71)	3,445 (79.45)	**0.77 (0.71, 0.83)**	**<0.001**

**History of diabetes, No. (%)**					
No	104,020 (94.12)	99,271 (94.52)	4,749 (86.41)	1 [Reference]	
Yes	6,502 (5.88)	5,755 (5.48)	747 (13.59)	**2.08 (1.92, 2.25)**	**<0.001**
**History of hypertension, No. (%)**					
No	28,906 (26.16)	28,202 (26.85)	704 (12.81)	1 [Reference]	
Yes	81,616 (73.85)	76,824 (73.15)	4,792 (87.19)	**1.67 (1.54, 1.81)**	**<0.001**
**History of hyperlipidemia, No. (%)**					
No	61,828 (55.94)	59,528 (56.68)	2,300 (41.85)	1 [Reference]	
Yes	48,694 (44.06)	45,498 (43.32)	3,196 (58.15)	**1.47 (1.39, 1.55)**	**<0.001**
**History of depression, No. (%)**					
No	104,485 (94.54)	99,358 (94.60)	5,127 (93.29)	1 [Reference]	
Yes	6,037 (5.46)	5,668 (5.40)	369 (6.71)	**1.57 (1.45, 1.70)**	**<0.001**
**LogMAR of visual acuity (better eye)**	-0.04 (0.15)	-0.05 (0.15)	-0.01 (0.16)	**1.97 (1.68, 2.31)**	**<0.001**

[a]Note: Cox proportional hazards regression models adjusted for age and gender.
ASCVD, atherosclerotic cardiovascular disease; No., number; HR, hazard ratio; CI, confidence interval；SD, standard deviation; LogMAR, logarithm of the minimum angle of resolution.

Supplementary Table 3. Sensitivity Analysis: Cox Proportional Hazards Models for Incident ASCVD by VA.[a]

	Model 1		Model 2	
	HR (95% CI)	P value	HR (95% CI)	P value
**Total analysis**				
**Visual acuity (Continue variable, per 0.1 LogMAR)**	1.85 (1.54, 2.22)	<0.001	1.50 (1.21, 1.87)	<0.001
**Visual acuity (Category variable)**				
T1 (high visual acuity level)	1 [Reference]		1 [Reference]	
T2 (moderate visual acuity level)	1.10 (1.02, 1.19)	0.014	1.00 (0.92, 1.10)	0.951
T3 (lowest visual acuity level)	1.26 (1.17, 1.36)	<0.001	1.13 (1.04, 1.23)	0.001
**P for trend**		<0.001		0.004
**Stratification analysis**				
**Stratified by gender**				
Female	1.93 (1.44, 2.58)	<0.001	1.48 (1.03, 2.12)	0.029
Male	1.80 (1.43, 2.28)	<0.001	1.51 (1.15, 1.98)	0.003
**Test for interaction**		0.348		0.620

[a] In sensitivity analysis, we removed all incident ASCVD cases that occurred within two years of follow-up to minimize the possibility of reverse causality.

We used Cox proportional hazards regression for the incident ASCVD. Model 1 was adjusted for baseline measurements of age and gender. Model 2 additionally adjusted for risk factors shared between VA and ASCVD, measured at baseline, including ethnicity, smoking status, education level, Townsend index, family history of severe ASCVD, physical activity level, and comorbidities (depression, diabetes, hypertension, and hyperlipidemia).

T, tertiles of LogMAR value.

VA, visual acuity; ASCVD, atherosclerotic cardiovascular disease; HR, hazard ratio; LogMAR, logarithm of the minimum angle of resolution.

Supplementary Table 4. Sensitivity Analysis: Cox Proportional Hazards Models for Incident ASCVD by VA. [a]

	**Model 1**		**Model 2**	
	HR (95% CI)	P value	HR (95% CI)	P value
**Total analysis**				
**Visual acuity (Continuous variable, per 0.1 LogMAR)**	1.97 (1.68, 2.31)	<0.001	1.65 (1.34, 2.03)	<0.001
**Visual acuity (Categorical variable)**				
T1 (high visual acuity level)	1 [Reference]		1 [Reference]	
T2 (moderate visual acuity level)	1.10 (1.03, 1.18)	0.005	1.00 (0.91, 1.08)	0.833
T3 (lowest visual acuity level)	1.30 (1.20, 1.37)	<0.001	1.16 (1.07, 1.26)	0.001
**P for trend**		<0.001		<0.001
**Stratification analysis**				
**Stratified by gender**				
Female	1.93 (1.49, 2.51)	<0.001	1.43 (1.00, 2.06)	0.052
Male	1.99 (1.62, 2.43)	<0.001	1.77 (1.37, 2.28)	<0.001
**Test for interaction**		0.725		0.819

[a] In sensitivity analysis, we further adjusted for clinically relevant variables, including systolic and diastolic blood pressure, HbA1c levels, LDL, HDL, and total cholesterol, to ensure that the findings remained consistent under additional levels of adjustment.

We used Cox proportional hazards regression for the incident ASCVD. Model 1 was adjusted for baseline measurements of age and gender. Model 2 additionally adjusted for risk factors shared between VA and ASCVD, measured at baseline, including ethnicity, smoking status, education level, Townsend index, family history of severe ASCVD, physical activity level, and comorbidities (depression, diabetes, hypertension, and hyperlipidemia), and clinically relevant variables (HbA1c levels, LDL, HDL, cholesterol, triglycerides).

T, tertiles of LogMAR value.

VA, visual acuity; ASCVD, atherosclerotic cardiovascular disease; HR, hazard ratio; LogMAR, logarithm of the minimum angle of resolution.

Supplementary Table 5. Mediation Analysis and Four-Way Decomposition of Associations of VA with ASCVD via Potential Mediators[a].

Measure	Overall		Women		Men	
	β	P Value	β	P Value	β	P Value
**Hypertension**						
Total effect	-0.497	<0.001	-0.452	0.002	-0.519	<0.001
Controlled direct effect	-0.365	<0.001	-0.278	0.005	-0.425	0.001
Reference interaction	-0.126	0.040	-0.163	0.205	-0.092	0.080
Mediated interaction	0.012	0.117	0.020	0.255	0.004	0.558
Pure indirect effect	-0.019	0.019	-0.031	0.015	-0.005	0.537
Proportion mediated, %	3.8	-	6.8	-	1.0	-
**Diabetes**						
Total effect	-0.452	<0.001	-0.320	0.029	-0.516	<0.001
Controlled direct effect	-0.245	<0.001	-0.278	<0.001	-0.202	0.034
Reference interaction	-0.198	0.031	-0.040	0.809	-0.303	0.012
Mediated interaction	0.005	0.052	0.000	0.810	0.012	0.035
Pure indirect effect	-0.015	<0.001	-0.009	0.026	-0.023	<0.001
Proportion mediated, %	3.3	-	2.8	-	4.5	-
**Depression**						
Total effect	-0.462	<0.001	-0.400	0.002	-0.493	<0.001
Controlled direct effect	-0.354	<0.001	-0.305	0.006	-0.380	0.001
Reference interaction	-0.088	0.361	-0.056	0.741	-0.103	0.398
Mediated interaction	0.006	0.366	0.005	0.741	0.004	0.418
Pure indirect effect	-0.026	<0.001	-0.040	<0.001	-0.014	0.007
Proportion mediated, %	5.7	-	10.0	-	2.6	-
**Townsend deprivation index**						
Total effect	-0.491	<0.001	-0.424	<0.001	-0.527	<0.001
Controlled direct effect	-0.467	<0.001	-0.387	0.003	-0.508	<0.001
Reference interaction	-0.013	0.785	-0.003	0.969	0.019	0.715
Mediated interaction	0.008	0.800	0.003	0.956	0.146	0.718
Pure indirect effect	-0.29	0.005	-0.037	0.009	-0.023	0.128
Proportion mediated, %	5.9	-	8.8	-	4.3	-

[a] Apart from the mediator being analyzed, other mediators were included as covariates to account for their potential influence on the mediator-outcome relationship. Additionally, the analysis adjusted for baseline measurements of age, gender, ethnicity, smoking status, education level, Townsend index, family history of severe ASCVD, physical activity level, and hyperlipidemia.

VA, visual acuity; ASCVD, Atherosclerotic Cardiovascular Disease;

Supplementary Table 6. Sensitive Analysis: Mediation Analysis and Four-Way Decomposition of Associations of VA with ASCVD via Potential Mediators[a].

Measure	Overall		Women		Men	
	β	P Value	β	P Value	β	P Value
**Hypertension**						
Total effect	-0.515	<0.001	-0.444	0.002	-0.551	<0.001
Controlled direct effect	-0.382	<0.001	-0.283	0.003	-0.451	<0.001
Reference interaction	-0.124	0.054	-0.143	0.324	-0.098	0.056
Mediated interaction	0.013	0.114	0.018	0.353	0.006	0.407
Pure indirect effect	-0.022	0.008	-0.036	0.009	-0.009	0.363
Proportion mediated, %	4.2	-	8.1	-	1.6	-
**Diabetes**						
Total effect	-0.483	<0.001	-0.394	0.003	-0.532	<0.001
Controlled direct effect	-0.234	<0.001	-0.256	<0.001	-0.200	<0.001
Reference interaction	-0.238	0.006	-0.129	0.373	-0.321	0.056
Mediated interaction	0.007	0.017	0.002	0.400	0.015	0.407
Pure indirect effect	-0.018	<0.001	-0.011	0.011	-0.027	0.363
Proportion mediated, %	3.7	-	2.8	-	5.0	-
**Depression**						
Total effect	-0.470	<0.001	-0.396	0.002	-0.505	<0.001
Controlled direct effect	-0.354	<0.001	-0.294	0.005	-0.389	<0.001
Reference interaction	-0.094	0.301	-0.065	0.692	-0.105	0.363
Mediated interaction	0.006	0.308	0.006	0.693	0.004	0.384
Pure indirect effect	-0.028	<0.001	-0.043	<0.001	-0.015	0.005
Proportion mediated, %	5.9	-	10.9	-	2.8	-
**Townsend deprivation index**						
Total effect	-0.528	<0.001	-0.465	<0.001	-0.563	<0.001
Controlled direct effect	-0.483	<0.001	-0.416	<0.001	-0.525	<0.001
Reference interaction	0.001	0.785	-0.007	0.931	0.006	0.906
Mediated interaction	0.001	0.800	0.006	0.899	-0.003	0.941
Pure indirect effect	-0.047	0.005	-0.058	<0.001	-0.040	0.007
Proportion mediated, %	8.9	-	12.4	-	7.1	-

[a]A sensitivity analysis was performed in which other mediators were excluded as covariates to assess the robustness of the results.

The analysis adjusted for age, gender, ethnicity, smoking status, education level, Townsend index, family history of severe ASCVD, physical activity level, and hyperlipidemia.

VA, visual acuity; ASCVD, Atherosclerotic Cardiovascular Disease;

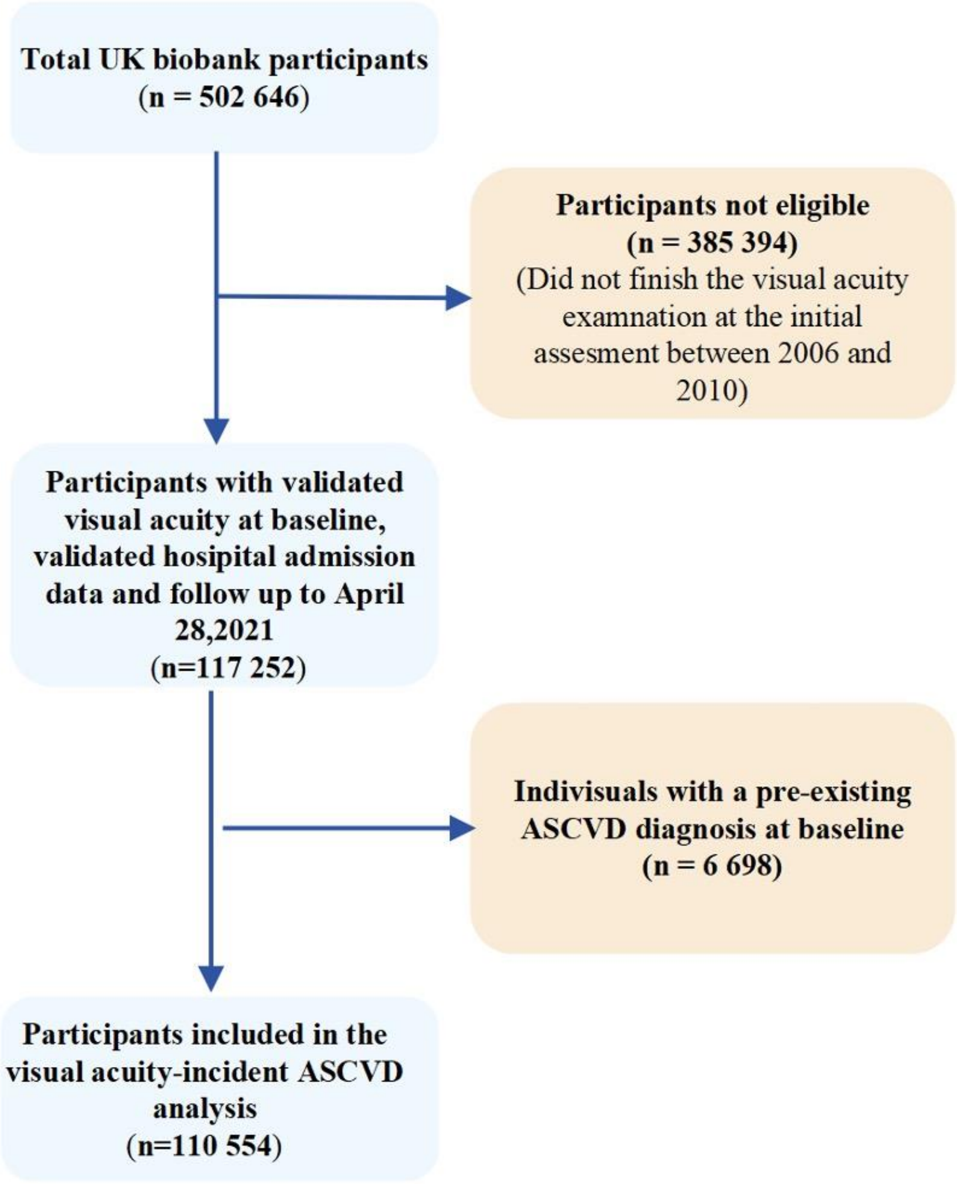

Supplementary Figure 1. Flow Chart of the Study Sample Selection from the UK Biobank.

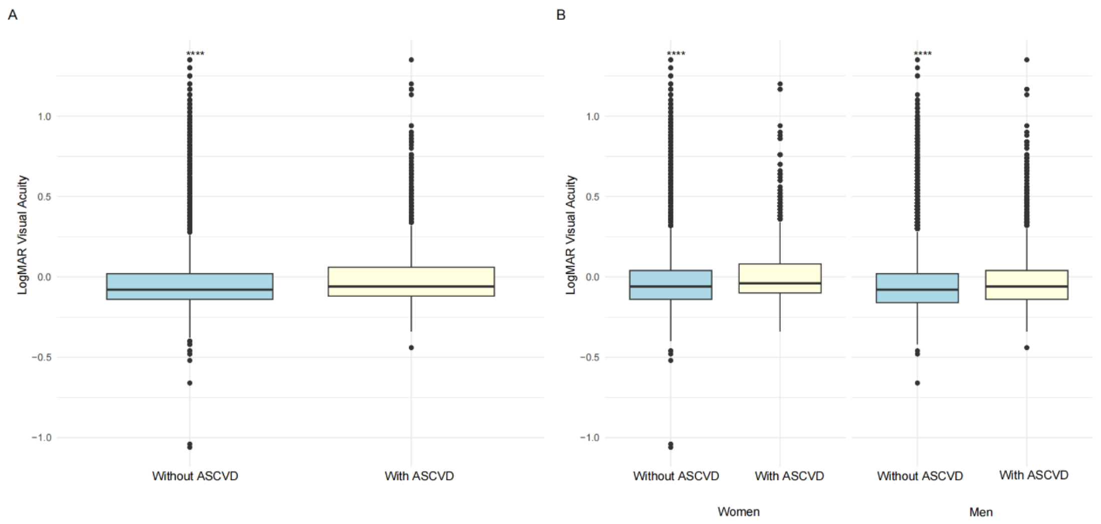

Supplementary Figure 2. Mean LogMAR Visual Acuity Stratified by Incident ASCVD.

(A) Total mean LogMAR values. (B) Sex-specific mean LogMAR values. Boxes contain 50% of the data, and the error bars contain the remainder. The horizontal lines indicate medians.

ASCVD, Atherosclerotic Cardiovascular Disease; LogMAR, logarithm of the minimum angle of resolution.

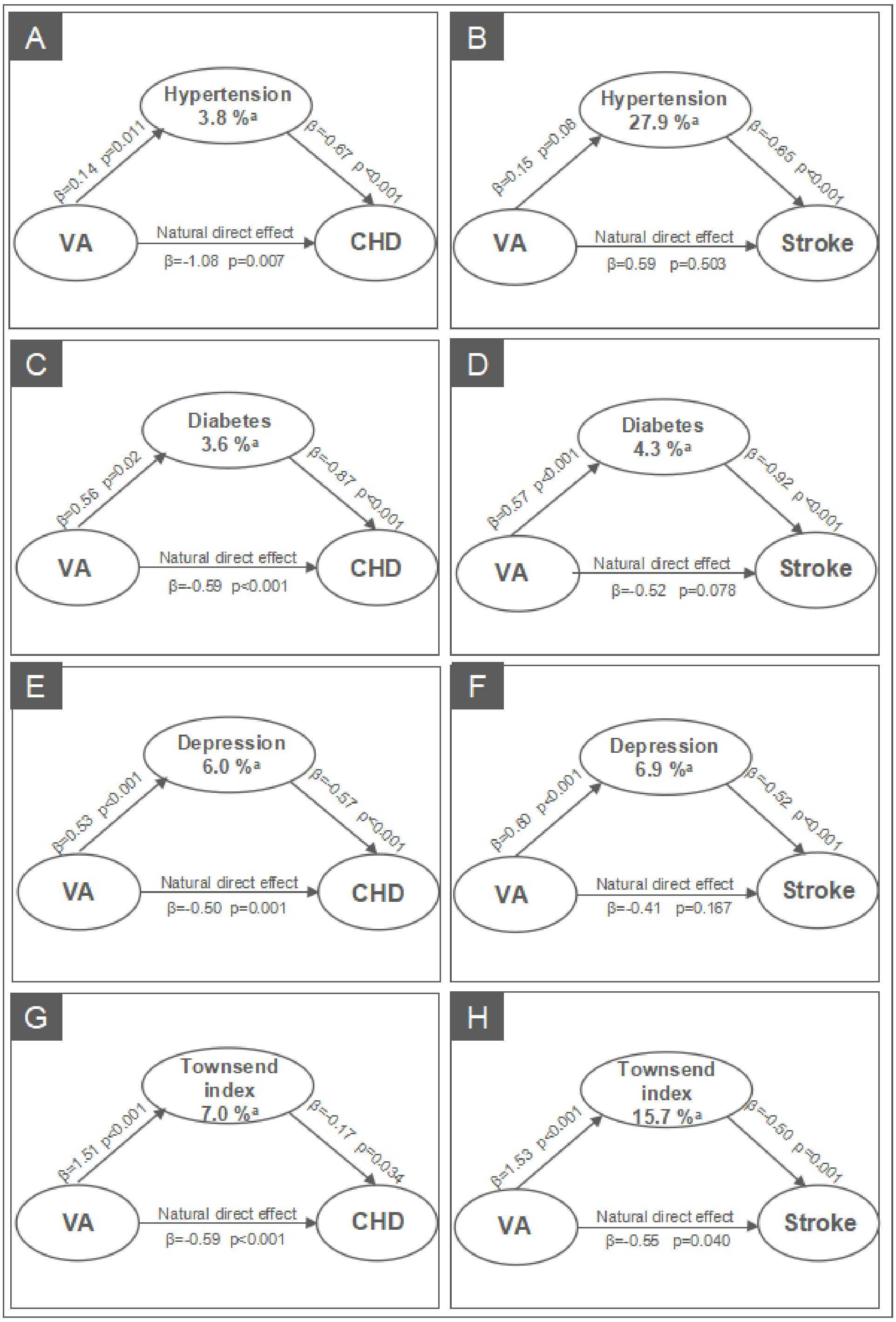

Supplementary Figure 3. Mediation Analysis for VA, CHD, and Stroke.

Mediation analyses were performed between VA-CHD for hypertension (panel A), for hypertension (panel C), for diabetes status (panel E) and for Townsend index (panel G) separately. Mediation analyses were performed between VA-Stroke for hypertension (panel B), for hypertension (panel D), for diabetes status (panel F) and for Townsend index (panel H) separately. Apart from the mediator being analyzed, other mediators were included as covariates to account for their potential influence on the mediator-outcome relationship. Additionally, the analysis adjusted for baseline measurements of age, gender,

ethnicity, smoking status, education level, Townsend index, family history of severe ASCVD, physical activity level, and hyperlipidemia. [a]P for indirect effect <0.05.

VA, visual acuity; ASCVD, atherosclerotic cardiovascular disease; CHD, coronary heart disease.
